# Editorial: Microbial Ecotoxicology Advances to Improve Environmental and Human Health Under Global Change

**DOI:** 10.3389/fmicb.2022.870404

**Published:** 2022-03-23

**Authors:** Aurélie Cébron, Dimitrios Georgios Karpouzas, Fabrice Martin-Laurent, Soizic Morin, Carmen Palacios, Mechthild Schmitt-Jansen

**Affiliations:** ^1^Université de Lorraine, CNRS, LIEC, F-54000 Nancy, France; ^2^Laboratory of Plant and Environmental Biotechnology, Department of Biochemistry and Biotechnology, University of Thessaly, Larissa, Greece; ^3^Agroécologie, INRAE, Institut Agro, Université de Bourgogne, Université de Bourgogne Franche-Comté, F-21000 Dijon, France; ^4^UR EABX, INRAE, F-33612 Cestas, France; ^5^Univ. Perpignan Via Domitia, CEFREM, UMR5110, F-66860, Perpignan, France; ^6^CNRS, CEFREM, UMR5110, F-66860, Perpignan, France; ^7^Helmholtz-Centre for Environmental Research—UFZ, Department of Bioanalytical Ecotoxicology, Leipzig, Germany

**Keywords:** microbial communities, impact of pollutants, environmental risk assessment, multiple stressors, biodegradation, bio-processes, ecosystem rehabilitation

Microbial Ecotoxicology is an interdisciplinary science at the intersection of microbial ecology, toxicology, ecotoxicology, and analytical chemistry (Ghiglione et al., 2016; Shahsavari et al., 2017; Pesce et al., 2020). This Research Topic was proposed aiming to present the full range of research currently in place in an emerging topic like microbial ecotoxicology. The research focus of microbial ecotoxicology spans from the assessment of the impact of various contaminants on microbial communities to the development of new bio-processes and includes studies on the microbial biodegradation of contaminants and rehabilitation of contaminated environments. In this Research Topic “*Microbial Ecotoxicology Advances to Improve Environmental and Human Health Under Global Change*,” we have collected 21 original articles presenting research on a range of contaminants (heavy metals, nanomaterials, biogenic and synthetic contaminants such as pesticides, herbicides, medicines, plastics and other agrochemicals). Presented research assesses their impacts on microbial diversity and activity, and their biodegradation. Contributions included the usage of microbial technologies to remove contaminants from contaminated environments and evaluation of essential microbial functions in contaminated and rehabilitated environments.

1. Microbial communities support several ecosystem functions and thus play a key role not only in biogeochemical cycles but also in a wide range of ecosystem services (Falkowski et al., 2008). Exposure of microbial communities to a range of contaminants can modify their abundance, composition and activity (Tang et al., 2019; Fei et al., 2020; Noyer et al., 2020) with consequences on the ecosystem functions they deliver as well as on higher levels of biological organization. In this Research Topic, changes in microbial community structure and functions under contamination stress were demonstrated in soils (Thiour-Mauprivez et al.), groundwater (Michel et al.), freshwater (Lyautey et al.; Kergoat et al.; Evariste et al.) and seawater (Cheng et al.). In Lake Geneva, the local anthropogenic contamination (organic matter, trace metals, PAH and PCB) induced lower bacterial and archaeal diversity but higher levels of many microbial activities (respiration, denitrification, methanogenesis, phosphatase and beta-glucosidase) and high abundance of antibiotic resistance genes (Lyautey et al.). Further, microbial activity (nitrification, Papadopoulou et al.; or denitrification, Michel et al.) was affected while functional population abundance was not impacted by synthetic pesticides. In other cases, the effect of antibiotics and graphene-based nanomaterials on the bacterial and diatom community composition, viability, physiology and interactions in biofilms were observed (Kergoat et al.; Evariste et al.). Unexpectedly, teratogenic effects of sulfonamide antibiotics on diatoms within periphyton were reported for the first time (Kergoat et al.). However, other studies reported no marked impact of other synthetic pesticide groups like ß-triketone herbicides toward bacterial communities (Thiour-Mauprivez et al.) or limited effects of plastic size and shape on the abundance, diversity and activity of bacterial communities on the plastic surfaces (Cheng et al.).
  Only few studies have addressed how cumulative stressors can alter ecosystem services. In this Research Topic, combined stressors studies have shown that ecosystems already stressed by the presence of contaminants may become more sensitive to additional environmental stresses and functional consequences amplified (Loustau et al.; Fikri et al.). The effect of droughts following a previous exposure to copper in biofilms, highlighted the importance of considering not only direct but also indirect effects of global changes (Loustau et al.). Moreover, low resistance but high resilience of an ecosystem after exposure to multiple stressors on already degraded soils was shown (Fikri et al.).
2. Due to their often-demonstrated capacities to transform and degrade a large range of substances including organic pollutants, microbial communities play a key role in the environmental fate of pollutants by regulating their persistence and mitigating related ecotoxicological impacts in the environment (Holliger et al., 1997; Haritash and Kaushik, 2009; Singh and Singh, 2016; Mohanan et al., 2020). Biodegradation of pesticides, medicines, plastics, antibiotics and foaming agents were studied in different environments to evaluate bioremediation and auto-depuration of impacted sites (Odobel et al.; Jacquin et al.; Hellal et al.; Crampon et al., Rolando et al.; Billet et al.). *In situ*, the microbial degradation of benzodiazepines was evaluated in soils (Crampon et al.) and the degradation of bio-based and fossil-based plastics in seawater was explored, where potential degraders were additionally identified (Odobel et al.). Transformation of the pesticide chlordecone and of two of its transformation products by microbial enrichment culture was assessed to identify degradation pathways, degradation key players and transformation products formed by microbial activities (Hellal et al.). The use of bacterial isolates or consortia capable of efficiently degrading organic contaminants such as antibiotics (Billet et al.) or foaming agents (Rolando et al.) in environmental matrices was explored. Finally, recommendations to improve future remediation strategies of polluted environments were proposed.
3. The cutting-edge research produced by microbial ecotoxicologists meets the demands of policy makers and the society in large. Specifically, it contributes to the tremendous challenges caused by intensive anthropogenic activities that threaten both environmental and human health worldwide. To bridge research to end-users, ecological engineering technologies are developed based on microbial technologies to support a more sustainable world. They aim to improve the management of contaminated environments and to bring up new treatment processes (Peng et al., 2018; Quintella et al., 2019; Bhatt et al., 2021). Here, new methods were developed to qualify and improve waste treatment and the quality of natural resources (Espinosa et al.; Anaya-Garzon et al.; Aigle et al.,; Haque et al.). First, a better management of water quality (such as cyanobacterial metabolites affecting the taste of drinking water) through the monitoring of microbial development was proposed (Espinosa et al.). Bacterial activities can help to treat and recover wastes, such as agricultural and urban organic wastes *via* anaerobic digestion (Aigle et al.), e-waste treatment through metal bioleaching (Anaya-Garzon et al.) or metal biosorption by bacterial biofilms (Haque et al.).
  Furthermore, after remediation or rehabilitation of contaminated sites, the recovery of the functioning of the rehabilitated ecosystem has to be assessed (Fikri et al.; Mghazli et al.). For example, the covering of acidic tailing with alkaline phosphate mine wastes was tested as a rehabilitation scenario of abandoned mines, and the status of microbial community diversity and functions were evaluated (Mghazli et al.). Nowadays, degraded urban soils can be ecologically rehabilitated by adding various materials to soils in order to restore the microbial functions involved in the C, P and N cycles (Fikri et al.).

Finally, emerging contaminants are constantly being introduced into the environment because of the implementation of new technologies in various industrial sectors and of the lack of prevention of possible contaminations issued from these new technologies. The fate and the impact of these emerging contaminants are often not well described and there is a need for monitoring. In particular, some environmental compartments are less monitored than others. For instance, the atmosphere is rarely considered in monitoring studies. In response to this, Samaké et al. demonstrated the importance of monitoring biogenic organic aerosol.

In summary, microbial ecotoxicology addresses several research challenges such as (i) providing an in-depth analysis of the changes imposed in the structure and functions of microbial communities under contamination, (ii) disentangling the complexity of environmental systems characterized of various interactors (toxicants, targets, multiple stressors), (iii) assessing the potential of contaminant biodegradation and utilization of the catabolic capacities of microbial communities for bioremediation of contaminated sites. Fundamental discoveries feed current applied developments including risk assessment using microbes in a changing world, development and validation of new methods to qualify environmental quality, guidelines for environmental policies and ecological engineering technologies based on microbial technologies. This Research Topic brings together original results concerning these challenging questions as well as articles addressing the latest advances in microbial ecotoxicology.

We are delighted to present this Research Topic in Frontiers in Microbiology. We hope that this e-book will be interesting and useful to the readers of Frontiers in Microbiology while highlighting the value of focusing on microbial ecotoxicology to broaden our knowledge on contaminants effect, biodegradation and treatment.

## Author Contributions

AC wrote the first version of the editorial manuscript. All authors contributed to manuscript write, revision, read, and approved the submitted version. All authors were co-editors of the Research Topic: *Microbial Ecotoxicology Advances to Improve Environmental and Human Health Under Global Change*.

## Conflict of Interest

The authors declare that the research was conducted in the absence of any commercial or financial relationships that could be construed as a potential conflict of interest.

## Publisher's Note

All claims expressed in this article are solely those of the authors and do not necessarily represent those of their affiliated organizations, or those of the publisher, the editors and the reviewers. Any product that may be evaluated in this article, or claim that may be made by its manufacturer, is not guaranteed or endorsed by the publisher.

## References

[B1] Bhatt P. Bhatt K. Sharma A. Zhang W. Mishra S. Chen S. (2021). Biotechnological basis of microbial consortia for the removal of pesticides from the environment. Critic. Rev. Biotechnol. 41, 317–338. 10.1080/07388551.2020.1853032 33730938

[B2] Falkowski P. G. Fenchel T. Delong E. F. (2008). The microbial engines that drive Earth's biogeochemical cycles. Science 320, 1034–1039. 10.1126/science.1153213 18497287

[B3] Fei Y. Huang S. Zhang H. Tong Y. Wen D. Xia X.. (2020). Response of soil enzyme activities and bacterial communities to the accumulation of microplastics in an acid cropped soil. Sci. Total Environ. 707, 135634. 10.1016/j.scitotenv.2019.135634 31761364

[B4] Ghiglione J. F. Martin-Laurent F. Pesce S. (2016). Microbial ecotoxicology: an emerging discipline facing contemporary environmental threats. Environ. Sci. Pollut. Res. 23, 3981–3983. 10.1007/s11356-015-5763-1 26578376

[B5] Haritash A. K. Kaushik C. P. (2009). Biodegradation aspects of polycyclic aromatic hydrocarbons (PAHs): a review. J. Hazard. Mater. 169, 1–15. 10.1016/j.jhazmat.2009.03.137 19442441

[B6] Holliger C. Gaspard S. Glod G. Heijman C. Schumacher W. Schwarzenbach R. P.. (1997). Contaminated environments in the subsurface and bioremediation: organic contaminants. FEMS Microbiol. Rev. 20, 517–523. 10.1111/j.1574-6976.1997.tb00334.x 9299718

[B7] Mohanan N. Montazer Z. Sharma P. K. Levin D. B. (2020). Microbial and enzymatic degradation of synthetic plastics. Front. Microbiol. 11, 2837. 10.3389/fmicb.2020.580709 33324366 PMC7726165

[B8] Noyer M. Reoyo-Prats B. Aubert D. Bernard M. Verneau O. Palacios c. (2020). Particle-attached riverine bacteriome shifts in a pollutant-resistant and pathogenic community during a Mediterranean extreme storm event. Sci. Total Environ. 732, 139047. 10.1016/j.scitotenv.2020.139047 32473395

[B9] Peng W. Li X. Xiao S. Fan W. (2018). Review of remediation technologies for sediments contaminated by heavy metals. J. Soils Sediments 18, 1701–1719. 10.1007/s11368-018-1921-718547718

[B10] Pesce S. Ghiglione J. F. Topp E. Martin-Laurent F. (2020). Editorial: Microbial Ecotoxicology. Front Microbiol. 20, 9. 10.3389/978-2-88963-881-9 PMC7333374 32676059

[B11] Quintella C. M. Mata A. M. Lima L. C. (2019). Overview of bioremediation with technology assessment and emphasis on fungal bioremediation of oil contaminated soils. Journal of environmental management 241, 156–166. 10.1016/j.jenvman.2019.04.019 30999265

[B12] Shahsavari E. Aburto-Medina A. Khudur L. S. Taha M. Ball A. S. (2017). “From microbial ecology to microbial ecotoxicology,” in Microbial Ecotoxicology (Cham: Springer), pp. 17–38

[B13] Singh B. Singh K. (2016). Microbial degradation of herbicides. Critic. Rev. Microbiol, 42, 245–261. 10.3109/1040841X.2014.929564 25159042

[B14] Tang J. Zhang J. Ren L. Zhou Y. Gao J. Luo L.. (2019). Diagnosis of soil contamination using microbiological indices: a review on heavy metal pollution. J. Environ. Manage, 242, 121–130. 10.1016/j.jenvman.2019.04.061 31028952

